# Chronic treatment with coenzyme Q10 mitigates the behavioral dysfunction of global cerebral ischemia/reperfusion injury in rats 

**DOI:** 10.22038/IJBMS.2022.57630.12865

**Published:** 2022-01

**Authors:** Iman Fatemi, Pooya Saeed Askari, Elham Hakimizadeh, Ayat Kaeidi, Sogand Esmaeil Moghaddam, Mohammad Pak-Hashemi, Mohammad Allahtavakoli

**Affiliations:** 1 Research Center of Tropical and Infectious Diseases, Kerman University of Medical Sciences, Kerman, Iran; 2 Student Research Committee, Rafsanjan University of Medical Sciences, Rafsanjan, Iran; 3 Physiology-Pharmacology Research Center, Research Institute of Basic Medical Sciences, Rafsanjan University of Medical Sciences, Rafsanjan, Iran; 4 Department of Physiology and Pharmacology, School of Medicine, Rafsanjan University of Medical Sciences, Rafsanjan, Iran

**Keywords:** Brain injuries, Free radicals, Ischemia/reperfusion, Q10, Rat

## Abstract

**Objective(s):**

The Ischemia/reperfusion (I/R) phenomenon has a critical role in brain injuries induced by some kinds of stroke. The current study investigates the effects of Coenzyme Q10 (Q10) on global cerebral I/R in rats.

**Materials and Methods:**

Fifty male Wistar rats were used in this study. The global cerebral I/R was induced by obstructing both common carotid arteries for 20 min and the animals were treated with Q10 (200 mg/kg; PO.) for 6 weeks. Depressive and anxiety-like behaviors were assessed using the elevated plus-maze and forced swimming test, respectively. Working and spatial learning and memory were assessed by the Y-maze continuous alternation task and Morris water maze. The brain tissues were evaluated for brain edema, brain-derived neurotrophic factor (BDNF) levels, and superoxide dismutase (SOD) activities.

**Results:**

Our results indicated that global cerebral I/R increased anxiety and depression-like behavior as well as reduced cognitive performance. Moreover, the levels of BDNF and activities of SOD are reduced in stroke animals. Chronic post-stroke treatment with Q10 decreased brain edema. Furthermore, Q10 administration reduced anxiety and depressive-like behavior as well as cognitive impairments in stroke animals. Q10 also increased the SOD activities and BDNF levels in the brain tissues of stroke animals.

**Conclusion:**

Finally, we can conclude that using Q10 supplementation may be beneficial against the global cerebral I/R complications.

## Introduction

The Ischemia/reperfusion (I/R) phenomenon has critical roles in brain injuries induced by stroke and trauma (1, 2). In this phenomenon, first, the blood flow of the brain decreases, which leads to ischemia, and reduction of toxic metabolites such as oxidative stress mediator elimination, and second, the blood flow of the brain increases, which leads to increased oxygen levels of the brain and overproduction of reactive oxygen species (ROS) (3, 4). Both of these processes (ischemia and reperfusion) are associated with over generation of free radicals which leads to cell membrane, protein, and DNA damages (5, 6). It is well established that I/R injuries induce anxiety, depression, and cognitive impairments (7-9). The global cerebral I/R model was used to mimic clinical conditions such as cardiac and cardiopulmonary arrest in which the blood flow of the brain is halted (1). Till now, there is no approved drug for the treatment of I/R stroke and many researchers tried to reduce the complications after insult.

Ubiquinone or Coenzyme Q10 (Q10) is a lipophilic vitamin-like substance that is an essential component of the electron transport chain of mitochondria and supports adenosine triphosphate biosynthesis (10). It is well established that Q10 possesses potent antioxidant effects. Previous reports demonstrated that Q10 could potentiate the capacity of the antioxidant defense enzymes (11). Moreover, Q10 protects neuronal cells via its antioxidant properties. On the other hand, Q10 could elevate the brain’s BDNF level as well as enhance the BDNF signaling pathway, which could explain the neuroprotective effects of this agent (12). The beneficial effects of Q10 have been demonstrated in various neurological diseases including Alzheimer’s and Parkinson’s diseases (13, 14). Also, the neuroprotective effects of Q10 against various models of stroke such as ischemic brain lesions induced by symptomatic vasospasm (15), photothrombotic model of stroke (16), and transient middle cerebral artery occlusion (17) have been investigated in previous reports. On the contrary, Li *et al*. showed that intraperitoneal injection of Q10 (3 mg/kg) during reperfusion and three hours after reperfusion did not inhibit neuronal injuries following global and focal ischemia (18). Moreover, Q10 administration (200 mg/kg for 8 weeks) mitigated venous ischemia/reperfusion injuries (19). Till now, no study has been performed on the effects of chronic post-stroke administration of Q10 in global cerebral I/R, and we decided to study the Q10 effects behaviorally and molecularly.

## Materials and Methods

***Animals***

Fifty male Wistar rats (190±10 g) were purchased from the animal house of Rafsanjan University of Medical Sciences (Rafsanjan, Iran) and housed under standard laboratory conditions with free access to food and water. All efforts were made to minimize pain and discomfort. All experiments were approved by and conducted under the Ethical Guidelines of the local ethical committee (ethical code: RUMS.REC.1399.148).

***Experimental groups and protocol***

After acclimatization for 2 weeks, the animals were divided into five identical groups as follows: (1) Control group: normal animals with no treatment; (2) Sham group: animals in this group underwent the same surgical procedures without the obstruction of arteries and any treatment; (3) Stroke group: animals in this group underwent global cerebral I/R induction with no treatment; (4) Stroke+Q10 group: stroke group was treated with Q10 (200 mg/kg/day; p.o.) for 6 weeks after stroke induction, and (5) Q10 group: normal animals treated with Q10 (200 mg/kg/day; p.o.) for 6 weeks.

Q10 (Zist Takhmir Co., Iran) was suspended in sodium carboxymethylcellulose (0.25% w/v) and given by a gavage needle once a day (volume: 10 ml/kg) (19). 

**Figure 1:**
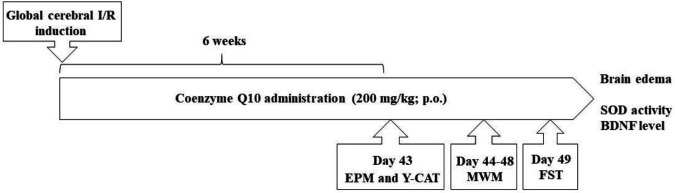
Schematic diagram of experimental design. I/R: Ischemia/reperfusion; P.O.: Per oral; EPM: elevated plus-maze; Y-CAT: Y-maze continuous alternation task; MWM: Morris water maze; FST: Forced swimming test; SOD: Superoxide dismutase; BDNF: Brain-derived neurotrophic factor

Figure 1 is a timeline diagram to facilitate understanding the procedure.

***Induction of global cerebral I/R***

Global cerebral I/R was induced as described in the literature (20). In brief, the animals were anesthetized through an IP injection of 10 mg/kg xylazine and 80 mg/kg ketamine (both from Alfasan Co., The Netherlands). Both common carotid arteries were exposed and the vagus nerves were separated from them. Then, both arteries were obstructed for 20 min by using the Yasargil aneurysm clips. 

***Behavioral tests***

*Elevated plus-maze (EPM)*

We used this method for evaluating anxiety-like behaviors (day 43). The maze consisted of two equal closed-arms (50×10×40 cm) and open-arms (50×10 cm) which connected to a central square platform (10×10 cm). The height of the maze from the floor was 50 cm. each animal was placed in the central platform with heads facing an open arm for 5 min. The percentages of open arms time (OAT%) and open arms entries (OAE%) were reported (21, 22). 

*Y-maze continuous alternation task (Y-CAT)*

We used this method for evaluating exploratory behavior and working memory (day 43). The maze consisted of three equal arms (40×4.5×12 cm). each animal was put in an arm and the frequency of entering each arm was manually recorded for 8 min. Correct alternation was defined as successive entries into a new arm before revisiting the two previously visited arms. The percentages of correct alternations [(frequency of correct alternations/total frequency of arm entries−2) × 100] were reported (23, 24).

*Morris water maze (MWM)*

We used this method for evaluating spatial learning and memory (days 44–48). The maze consisted of a black circulation pool (diameter: 120 cm and height: 40 cm) and a black circulation platform (diameter: 10 cm). The pool was filled with freshwater and divided into four identical quadrants. The platform was placed in the center of the target quadrant 2 cm below the water. To help the animals find the platform, pieces of paper with various shapes were fixed on the wall of the testing room, as a marker. The animals had four trials per day for four successive days. Each animal was put into the water from one of four different quadrants. In case the animals did not locate the platform within the cut-off time (120 sec), they were led to it by the examiner and stayed there for 15 sec. After 24 hr, the concealed platform was removed, and the animals were put into the maze. The escape latency, swimming speed, number of crossings through the concealed platform location, and the time spent in the target quadrant were reported (25). 

*Forced swimming test (FST)*

We used this method for evaluating depressive-like behaviors (days 49). The test consisted of a cylindrical pool (diameter of 20 cm and height of 55 cm) filled with freshwater (34±1 °C) to a depth of 45 cm. Each animal was put into the pool for 5 min. The immobility time (floating in the water without struggling) was reported (23). 

*Tissue preparation*

on the 50^th^ day of the experiment, the rats were decapitated and the brains were immediately harvested. In each group, five brain samples were used to evaluate brain edema. Both cerebral hemispheres of the other five brain samples were homogenized (1:10 w/v) in ice-cold sterile PBS (phosphate-buffered saline) and centrifuged (16,000 × rpm at 4 °C for 20 min) to collect the supernatants and evaluate the BDNF level and SOD activity (26, 27). The protein concentration was evaluated by the Bradford method.

*Assessment of the brain edema*

The content of brain water was used for evaluating brain edema. At first, the brains were weighed (wet weight), kept in an autoclave (at 60–70 °C for 72 hr), and weighed again (dry weight). The percentage of brain edema was calculated using the following formula (28):

The percent of brain edema = ([dry weight – wet weight]/wet weight) × 100

*Assessment of BDNF levels and SOD activities*

BDNF levels were evaluated with an ELISA kit (ZB-10013S-M9648, Zellbio Co., Germany), according to the manufacturer’s guidelines. Briefly, the samples were defrosted and put in the kit wells. Then, the conjugated antibody was added to the wells and incubated on the shaker for 45 min at room temperature. In the next stage, the wells were washed by using a washer buffer to remove free antibodies or antigens. After washing, the substrate solution was added to the wells and incubated for 15 min. At last, the interrupter solution was added to wells and light absorption was read at 450 mm (RT-2100C, Rayto, China). Results were expressed as pg/mg protein (29).

SOD activities were measured using an assay kit (ZB-10290S-M9648, Zellbio Co., Germany), according to the manufacturer’s guidelines. Briefly, the samples were defrosted, added to the chromogen solution, and shaken well. Then, light absorbance was read at 420 nm (RT-2100C, Rayto, China). Results were expressed as U/mg protein (30).

***Statistical analysis***

Results were reported as the mean ± SEM. Repeated measurement ANOVA (RMA) followed by the Tukey test was used for comparison of the escape latency between different groups in the MWM test. Other comparisons were performed using one-way ANOVA followed by Tukey’s test. The value of *P*<0.05 was represented statistically significant.

## Results

***Brain edema***

**Figure 2:**
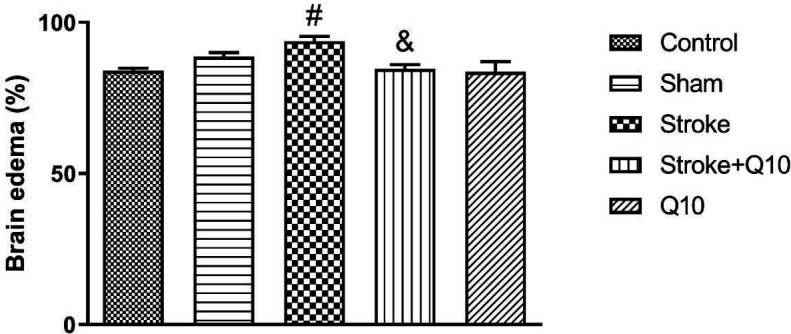
Percentage of brain edema in experimental groups of rat. Data were presented as mean ± SEM (n=5). # *P*<0.05 significant difference between sham and stroke groups. & *P*<0.05 significant difference between stroke and stroke+Q10 groups. Q10: Coenzyme Q10

Induction of stroke increased the percentage of brain edema compared with the sham group (*P*<0.05) (Figure 2). In addition, treatment with Q10 decreased the percentage of brain edema in comparison with the stroke group (*P*<0.05). Furthermore, it was shown that Q10 alone has no effect on this index in healthy animals.

***Behavioral tests***

*EPM*

**Figure 3:**
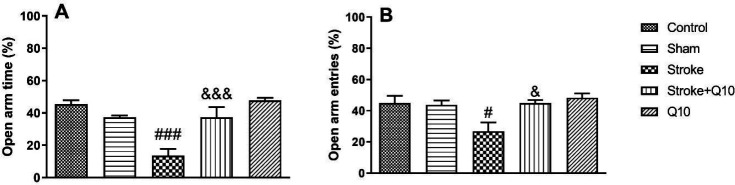
Elevated plus-maze tests in experimental groups of rat. Data were presented as mean ± SEM (n=10). # *P*<0.05 and ## *P*<0.001 significant difference between sham and stroke groups. & *P*<0.05 and &&& *P*<0.001 significant difference between stroke and stroke+Q10 groups. Q10: Coenzyme Q10

Stroke induction reduced OAT% and OAE% compared with the sham group (*P*<0.001 and *P*<0.05, respectively) (Figures 3A and B). In addition, chronic treatment with 200 mg/kg Q10 increased OAT% and OAE% compared with the stroke group (*P*<0.001 and *P*<0.05, respectively). Furthermore, it was shown that Q10 alone has no effect on these indices in healthy animals.

*Y-CAT*

**Figure 4:**
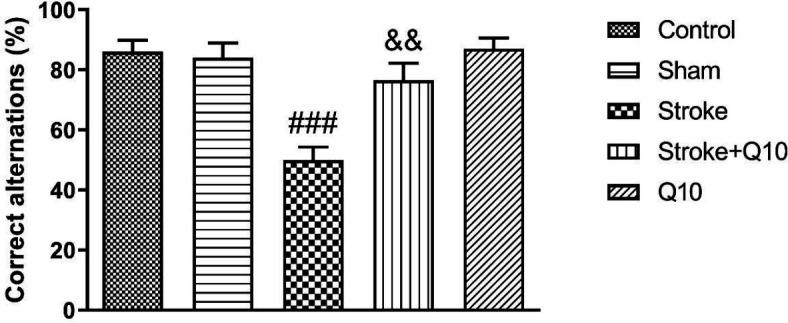
Percentage of correct alternations in experimental groups of rat. Data were presented as mean ± SEM (n=10). ### *P*<0.001 significant difference between sham and stroke groups. && *P*<0.01 significant difference between stroke and stroke+Q10 groups. Q10: Coenzyme Q10

Results from Y-CAT showed a significant difference in the percentage of correct alternations of the stroke group compared with the sham group (*P*<0.001) (Figure 4). In addition, treatment with 200 mg/kg Q10 increased the percentage of correct alternations compared with the stroke group (*P*<0.01). Furthermore, it was shown that Q10 alone has no effect on this index in healthy animals.

*MWM*

**Figure 5:**
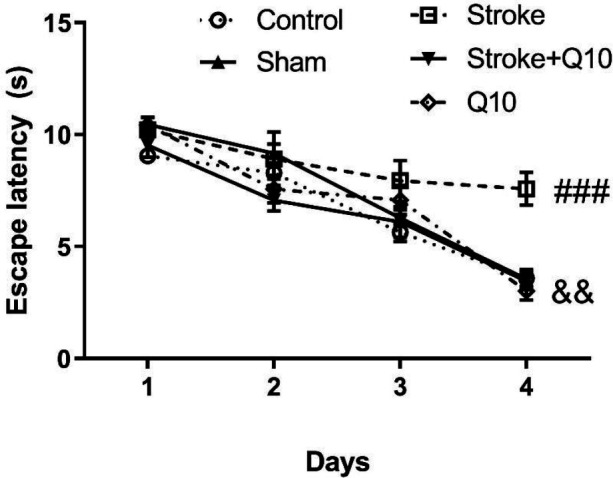
Escape latency in experimental groups of rat. Data were presented as mean ± SEM (n=10). ### *P*<0.001 significant difference between sham and stroke groups. && *P*<0.01 significant difference between stroke and stroke+Q10 groups. Q10: Coenzyme Q10

The results of MWM revealed that induction of stroke increases the escape latency compared with the sham group (RMA, *P*<0.001) (Figure 5). In addition, treatment with 200 mg/kg Q10 reduced the escape latency compared with the stroke group (*P*<0.01). Stroke induction reduced the time spent in the target quadrant in comparison with the sham group (*P*<0.05) (Table 1). Treatment with 200 mg/kg Q10 increased the time spent in the target quadrant compared with the stroke group (*P*<0.001). Furthermore, the number of crossings through the hidden platform position was decreased in the stroke group in comparison with the sham animals (*P*<0.01) (Table 1). Treatment with Q10 increased the number of crossings compared with the stroke group (*P*<0.01). Also, our data revealed that the velocity did not change significantly in different experimental groups (Table 1). Furthermore, it was shown that Q10 alone has no effect on these indices in healthy animals.

*FST*

**Figure 6:**
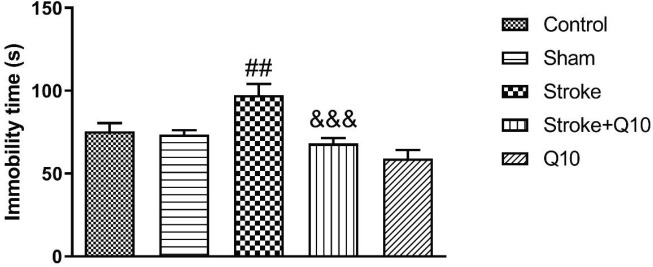
Immobility time in experimental groups of rat. Data were presented as mean ±SEM (n=10). ## *P*<0.01 significant difference between sham and stroke groups. &&& *P*<0.001 significant difference between stroke and stroke+Q10 groups. Q10: Coenzyme Q10

Stroke induction increased the immobility time compared with the sham group (*P*<0.01) (Figure 6). In addition, treatment with Q10 (200 mg/kg) reduced the immobility time in comparison with the stroke group (*P*<0.001). Furthermore, it was shown that Q10 alone has no effect on this index in healthy animals.

*BDNF Levels and SOD Activities*

**Figure 7:**
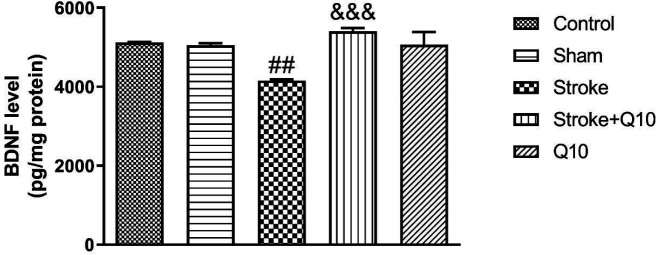
Levels of BDNF in experimental groups of rat. Data were presented as mean ± SEM (n=5). ## *P*<0.01 significant difference between sham and stroke groups. &&& *P*<0.001 significant difference between stroke and stroke+Q10 groups. Q10: Coenzyme Q10

The levels of BDNF were reduced in the stroke animals compared with the sham group (*P*<0.01) (Figure 7). In addition, treatment with Q10 (200 mg/kg) increased the levels of BDNF compared with the stroke group (*P*<0.001). 

**Figure 8:**
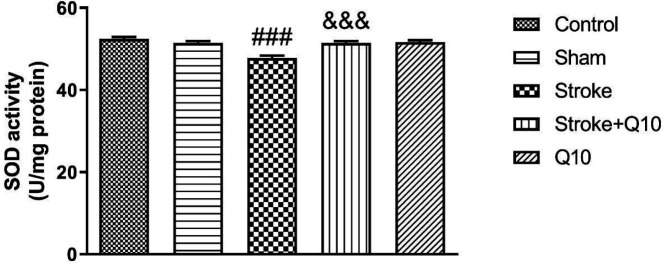
Activities of superoxide dismutase (SOD) in experimental groupsof rat. Data were presented as mean ± SEM (n=5). ### *P*<0.001 significant difference between sham and stroke groups. &&& *P*<0.001 significant difference between stroke and stroke+Q10 groups. Q10: Coenzyme Q10

Our data indicated that induction of stroke reduced the activities of SOD compared with the sham group (*P*<0.001) (Figure 8). In addition, treatment with Q10 (200 mg/kg) significantly decreased the activities of SOD compared with the stroke group (*P*<0.001). 

Furthermore, it was shown that Q10 alone has no effect on these indices in healthy animals.

**Table 1:** Morris water maze tests in experimental groups of rat

	Control	Sham	Stroke	Stroke+Q10	Q10
**Time in the target quadrant (s)**	12.72±0.31	12.47±0.21	9.43±0.67^#^	18.33±1.18^&&&^	12.25±0.42
**Number of crossings (n)**	5.70±0.33	5.50±0.34	3.70±0.26^##^	5.30±0.26^&&^	5.30±0.33
**Velocity (cm/min)**	30.13±0.40	28.39±0.95	28.57±0.58	28.25±1.01	29.59±0.55

Data were presented as mean ± SEM (n=10)

# *P*<0.05 and ## *P*<0.01 significant difference between sham and stroke groups.

&& *P*<0.01 and &&& *P*<0.001 significant difference between stroke and stroke+Q10 groups

Q10: Coenzyme Q10

## Discussion

In cerebral I/R, the restoration of blood flow induced severe injuries to insulted neurons (31). The exact mechanism of this phenomenon is not fully clear. Different mechanisms are involved and the most common and well-investigated factors are oxidative stress and edema (32). According to our results, chronic post-stroke treatment with Q10 (200 mg/kg) could significantly reduce brain edema and attenuate cognitive impairments, while decreasing depressive and anxiety-like behavior and increasing BDNF levels as well as SOD activity after global cerebral I/R injury in rats.

We found that the percentage of brain edema in animals of the stroke group is higher than that of the control animals. Also, we demonstrated that chronic post-stroke treatment with Q10 (200 mg/kg) decreased brain edema in global cerebral I/R rats. Brain I/R injury is associated with blood-brain barrier (BBB) disruption which leads to brain edema via increasing the permeability of brain capillaries. The protective effects of Q10 on BBB have not yet been investigated and this is the first report in this regard. Q10 is an antioxidant and it was shown that different anti-oxidants such as vitamin C (33), naringin (34), and kaempferol (35) reduced the permeability of BBB in ischemic injuries. Accordingly, it seems that Q10 decreases BBB rapture and brain edema via its potent antioxidant activities.

In both humans and animals, ischemic stroke-induced psychiatric problems such as anxiety and depression are associated with functional impairment. It is well established that global cerebral I/R induce anxiety and depression through increasing the brain oxidative stress. Our results also indicated that in stroke animals depressive and anxiety-like behaviors increased. Moreover, we demonstrated that administration of Q10 to stroke animals reduces depressive and anxiety-like behaviors. In agreement with our results, the anti-depressive and anxiolytic effects of Q10 have been demonstrated in previous reports. Onaolapo *et al*. found that Q10 supplement (60 mg/kg for 21 days) decreases anxiety-related behaviors in parkinsonism animals (36). Furthermore, Ibrahim Fouad revealed that Q10 reduced depressive behavior in rats with Alzheimer’s disease through neuroprotective properties (37). 

BDNF has an important role in cognitive functions via direct and/or indirect activities such as neuroprotective effects via reducing neuronal apoptosis and atrophy as well as modulating synaptic plasticity (38, 39). It is well documented that cognitive impairments in some neurological diseases are associated with reduction of BDNF levels (40). Our results also revealed that the levels of BDNF decreased in stroke animals. Moreover, we demonstrated that treatment with Q10 (100 mg/kg) before and after stroke significantly increased BDNF levels in stroke animals.

In agreement with the current investigation, the enhancing properties of Q10 on the level of BDNF were supported by previous reports. A study showed that Q10 increased the cortical and hippocampal levels of BDNF in phenytoin-treated rats (12). Recently, it has been shown that chronic treatment with Q10 (100 mg/kg for 6 weeks) reduced the depressive-like behaviors in animals exposed to chronic unpredictable mild stress via elevating BDNF levels (41). Q10 may have exerted these beneficial properties against cerebral I/R through the neuroprotective effects of BDNF.

The I/R phenomenon in the brain led to overgeneration of ROS and reduced the capacity of the antioxidative defense system (42-44). Decreasing the levels and/or activities of antioxidant enzymes such as SOD as well as increasing the levels of ROS is associated with some of the behavioral manifestations of stroke including cognitive disorders, depression, and anxiety (44, 45). We also demonstrated that global cerebral I/R decreased the activity of SOD in the brain tissue. Our results also revealed that chronic post-stroke treatment with Q10 (200 mg/kg) increased the brain SOD activity in the stroke group. Q10 has potent antioxidant activity through direct and/or indirect properties. It is well established that Q10 as an endogenous antioxidant could directly inactivate the free radicals (11). On the other hand, this agent could show its antioxidant activity by increasing the capacity of the antioxidant system. Researchers found that pre-treatment with Q10 (40 mg/kg for 1 week) reduced the cerebral ischemia complications via increasing the activity of SOD (46). Moreover, it was demonstrated that Q10 attenuated sevoflurane-induced neuroinflammation by increasing the SOD level (47). Accordingly, Q10 might attenuate the behavioral complications of global cerebral I/R through enhancement of the SOD activity.

It is confirmed that I/R conditions in the brain induced cognitive impairments such as reduced learning and memory that may be attributable to the death of brain neurons (48, 49). The results presented here also indicated the deleterious effects of global cerebral I/R on cognitive performance. We found that global cerebral I/R decreases the working memory in Y-CAT and spatial learning and memory in MWM. Moreover, we observed that chronic post-stroke administration of Q10 (200 mg/kg) attenuates these deleterious effects of stroke on cognitive functions. The protective effects of Q10 on cognitive function have been found in different pathological conditions. For example, a study showed that Q10 reduced the disorders of learning and spatial memory in hippocampal injury of mice via antiapoptotic and neuroprotective effects (50). In another study, it was demonstrated that Q10 reduced cognitive dysfunction in an animal model of Alzheimer’s disease via reducing oxidative stresses and inflammation (13). As above mentioned, Q10 could increase the brain levels of BDNF following global cerebral I/R, and BDNF as an important growth factor in the brain has a critical function in cognitive processes (38, 39). Hence, Q10 may mitigate the cognitive impairments of global cerebral I/R via different neuroprotective mechanisms such as decreasing inflammation and oxidative stress as well as increasing BDNF.

## Conclusion

Overall, the results of the current investigation revealed the potential protective effects of chronic post-stroke administration of Q10 on global cerebral I/R injury associated with the reduction of brain edema, cognitive impairments as well as anxiety and depressive-like behavior. Moreover, Q10 increased the SOD activity and BDNF level in the brain tissue of stroke animals. Thus, the new findings of the present research suggest Q10 as a therapeutic candidate against global cerebral I/R brain injury. Further investigations for answering any queries regarding the neuroprotective mechanism of Q10 against ischemia must be conducted. 

## Authors’ Contributions

MA and IF conceived and designed the experiments; PSA, SEM, and MPH performed the experiments. EH and AK analyzed the data. AK, IF, and MA contributed reagents/materials/analysis tools. IF and MA wrote the paper. All authors read and revised the manuscript.

## Funding Information

This paper was based on the thesis of a medical student (Pooya Saeed-Askari) and was supported by a grant from the Vice-Chancellor for Research and Technology, Rafsanjan University of Medical Sciences, Rafsanjan, Iran (grant number: 98065).

## Conflicts of Interest

The authors declare that there are no conflicts of interest regarding the publication of this article.
